# Powder milk as a user-friendly tool for baked milk challenge

**DOI:** 10.1186/1710-1492-10-S1-A33

**Published:** 2014-03-03

**Authors:** Sabrine Cherkaoui, Louis Paradis, Philippe Bégin, Anne Des Roches

**Affiliations:** 1 Division of Internal Medicine, Department of Medicine, University of Montreal, Montreal, QC, Canada; 2 Divisions of Pediatric Allergy and Immunology, Department of Pediatrics, Sainte-Justine Hospital, Montreal, QC, Canada; 3 Divison of Clinical Immunology and Allergy, Department of Medicine, University of Montreal, QC, Canada

## Background

Cow’s milk allergy is the most common food allergy among children. Previous studies have reported that up to 75% of children may tolerate baked milk goods. Various forms of baked milk challenges have been used in the literature such as muffins, pizza, and waffles. However, the food used for baked milk challenge is often prepared in a non-standardized manner by the parents at home, raising concerns with regards to validity, reproducibility and convenience. Instant skim milk powder is made by a food process that involves heating skim milk to up to 200°C for up to 30 minutes which should be sufficient to denature thermo-labile proteins.

## Objective

To evaluate the usefulness of skim milk powder as a convenient standardized form of baked milk challenge.

## Methods

All challenges to instant skim milk powder (cumulative dose of 4g proteins) performed at Sainte-Justine Hospital in Montreal, Canada between November 2008 and January 2013 were retrospectively reviewed. Observed reaction rates to challenge and to subsequent home introduction were compared to previous literature using different forms of baked milk. Demographic data, clinical characteristics, skin prick tests (SPT) and specific IgE levels were compared between those that passed and those that failed the challenge.

## Results

Thirty-nine children underwent an open food challenge to instant skim milk powder and thirty patients (76,9%) passed the challenge. All of those who passed the challenge successfully introduced baked milk products at home. Compared to those who were baked milk tolerant, baked milk reactive children had higher median specific IgE levels to cow’s milk (P < .0005), casein (P < .001), α-lactalbumin (P < .001) and β-lactoglobulin (P < .04). Both cohort reaction rates and characteristics were comparable to previous literature using other forms of baked milk product for challenge.

## Conclusion

Challenge with instant skim milk powder is a safe, convenient and easily standardized alternative to home baked food for baked milk challenge.

